# Altered Expression of ZnT10 in Alzheimer's Disease Brain

**DOI:** 10.1371/journal.pone.0065475

**Published:** 2013-05-31

**Authors:** Helen J. Bosomworth, Paul A. Adlard, Dianne Ford, Ruth A. Valentine

**Affiliations:** 1 Human Nutrition Research Centre, Newcastle University, Newcastle upon Tyne, United Kingdom; 2 School of Dental Sciences, Newcastle University, Newcastle upon Tyne, United Kingdom; 3 Institute for Cell and Molecular Biosciences, Newcastle University, Newcastle upon Tyne, United Kingdom; 4 The Florey Institute of Neuroscience and Mental Health, The University of Melbourne, Melbourne, Australia; Alexander Flemming Biomedical Sciences Research Center, Greece

## Abstract

There is an increasing body of evidence suggesting that metal homeostasis is dysregulated in the pathology of Alzheimer's disease (AD). Although expression levels of several transporters belonging the SLC30 family, which comprises predominantly zinc transporters, have been studied in the AD brain, SLC30A10 (ZnT10) has not been studied in this context. To determine if dysregulated expression of ZnT10, which may transport both Zn and Mn, could be a factor that contributes to AD, we investigated if there were differences in ZnT10 mRNA levels in specimens of frontal cortex from AD patients and controls and also if brain tissue from the APP/PS1 transgenic (Tg) mouse model showed abnormal levels of ZnT10 mRNA expression. Our results show that ZnT10 is significantly (*P<*0.01) decreased in the frontal cortex in AD. Furthermore, we observed a significant decrease in ZnT10 mRNA levels in the APP/PS1-Tg mice compared with wild-type controls (*P<*0.01). Our results suggest that this dysregulation in ZnT10 could further contribute to disease progression.

## Introduction

Zn is thought to be involved in Alzheimer's disease (AD) development and progression, however there are many questions that remain unanswered. It is evident that the transcription of amyloid precursor protein (APP) is regulated by the Zn-dependent transcription factors Sp1 and NF-κB [1]. In addition, Zn is able to promote formation of amyloid-β (Aβ) oligomers and aggregates at physiological concentrations available in the brain by binding Aβ at 3 N-terminal histidine residues [2]. Moreover, the protease responsible for APP cleavage, α-secretase, is inhibited by Zn [2]. It follows that dysregulation of mechanisms that ordinarily act to maintain Zn homeostasis within brain tissue may contribute to AD. The possible involvement of selected Zn transporters in AD has been investigated already, but more comprehensive study in this area is warranted. Regulation of Zn is maintained through two families of Zn transporters; ZnTs and ZIPs. Human studies of Zn transporter dysregulation in AD have focussed on the ZnTs, with regulation of ZnT1, 3, 4, 6 and 7 being studied previously. Up-regulation of ZnT1 protein in the hippocampus in AD brain tissue has been observed [3]. It is thought that this increase in ZnT1 expression in the disease state causes an increase in Zn ions available in the extracellular space for initiation of Aβ deposition and senile plaque formation [4]. In the preclinical disease states - mild cognitive impairment and pre-clinical AD- an initial decrease in ZnT1 is also observed before progression to AD. Positive correlation between Braak score (NFT pathology) and ZnT1 levels has also been reported [2], [4]. Additional members of the ZnT family of Zn transporters, ZnT4 and ZnT6, have also been implicated in AD progression, with elevated protein levels of both transporters in the hippocampus of AD patients [5].

It has been suggested that ZnT6, located in the Golgi apparatus, mediates accumulation of Zn therefore enabling Zn to promote Aβ formation by binding to APP and inhibiting α-secretase [4]. ZnT4 is found in the lysosomal and endosomal compartments in hippocampal tissue, where an increase in expression observed in AD patients [5] may drive the sequestration of Zn, consistent with Aβ accumulation in these vesicles in the post-mortem AD brain [6].

ZnT3 localises predominantly to the membranes of Zn-rich synaptic vesicles in the hippocampus [7], and Znt3 knockout mice are devoid of Zn in these otherwise Zn-enriched terminals. Furthermore, increased amyloid deposition in the brain of female mice is co-incident with an age-dependent hyperactivity of the Znt3 protein in the female Tg2576 mouse model compared with the male mice, which is abolished in the Znt3 knockout mouse [8]. This result is particularly interesting in light of the higher incidence of AD in women compared with men. In addition, in the Tg2576 mouse model of AD the ablation of Znt3 inhibits the development of Aβ pathology. The levels of mRNA and protein expression of ZnT3 appear to be lower however, in the cortical regions of human AD patients [9], [10], in contrast to the mouse model where Znt3 protein levels are increased [11].

Manganese (Mn) is another trace element that is essential in the body. It is an important co-factor for several enzymes in the brain, including Mn-superoxide dismutase and glutathione synthetase. It is also needed for adequate neurotransmitter synthesis and metabolism [12]. As with Zn homeostasis, Mn levels in the body are tightly controlled, with several manganese transporter systems being characterised. Intriguingly other Zn transporters (ZIP8 and ZIP14) have been associated with Mn transport, although their levels of expression in the brain are lower than in other tissues [13]–[16]. High levels of Mn in the brain have been associated with dysfunction of the basal ganglion system that can cause severe neurological disease [13] with chronic exposure leading to a motor syndrome resembling idiopathic Parkinson's disease, characterised by the presence of Alzheimer's Type II astrocytosis [12]. We and others have recently identified relatively high levels of a further member of the ZnT family (SLC30;based upon homology with other ZnT family members), ZnT10, in brain tissue [17], [18]. We have also discovered that extracellular Zn treatment down regulates ZnT10 expression at both the mRNA and protein levels and, using an indirect method, measured a functional response to ZnT10 overexpression commensurate with reduced intracellular zinc availability [17]. Interestingly recent studies have indicated that ZnT10 may primarily transport Mn, rather than Zn. These assumptions are based upon the fact that mutations in the ZnT10 protein impair manganese homeostasis [18] and that a Mn-sensitive yeast strain can be rescued by expression of human ZnT10 but not by a mutated versions, consistent with it ZnT10 acting as a manganese transporter [19], [20]. Furthermore recent studies have revealed two different homozygous frame shift mutations in ZnT10 and have implicated these mutations with neurological disorders, including adult onset Parkinsonism and juvenile onset dystonia [18].

Further understanding of the ZnT family and their interactions in the brain may elucidate a role for ZnTs and Zn and/or Mn in the progression and pathology of AD. To date ZnT10 has not been studied in this context. To determine if dys-regulated expression of ZnT10 could be a factor that contributes to AD, we investigated if there were differences in ZnT10 mRNA levels in specimens of frontal cortex from AD patients and controls and also if brain tissue from the APP/PS1 transgenic (Tg) mouse model showed abnormal levels of ZnT10 mRNA expression.

## Materials and Methods

### Ethics statement

All human tissue was obtained from the Newcastle Brain Tissue Resource at Newcastle University which is a Human Tissue Authority licensed Research Tissue Bank following ethical approval by the National Research Ethics Service. All donations were obtained with fully informed consent following an NRES approved protocol.

All mouse experiments were carried out in accordance with the local animal ethics committee requirements (Howard Florey Animal Ethics Committee) and National Health and Medical Research Council standards of animal care (Australia).

### Brain specimens

Frontal cortex RNA samples from human Alzheimer's disease pathology cases (*n* = 16; female *n* = 10, male *n* = 6) and age-matched controls (*n* = 11; female *n* = 4, male *n* = 7; Table 1. Individual data available in Table S1). For mouse samples, frontal cortex brain tissue samples were obtained from 12 month old female wild-type (WT) (*n* = 5) and APP/PS1 mice (*n* = 11). APP/PS1 mice are a mouse model of AD containing two transgenes inserted at a single locus that develop beta-amyloid deposits in the brains by 6 to 7 months of age (http://jaxmice.jax.org/strain/005864.html). Brain samples were removed and snap frozen before being stored in RNA Later at −80°C.

**Table 1:** Subject demographic data.

Group	Age (years, mean ± SEM)	PM Delay (mean ± SEM)	Braak staging (median)
Control	71±8.0 (n = 11; female = 4, male = 7)	20.8±3.0	II
AD	78±2.2 (n = 16; female = 10, male = 6)	15.4±1.8	V

### RNA extraction and RT-qPCR

Total RNA was extracted from brain tissue using TRIzol® reagent. Concentration of RNA was determined by measuring the absorbance on a Nano drop ND-1000 (NanoDrop®). Total RNA preparations were analysed using an Agilent 2100 Bioanalyzer (Agilent Technologies). Samples were stored at −80°C. RNA was DNAse treated and synthesis of RNA into cDNA was carried out using M-MLV RT (Promega) according to manufacturer's instructions in 25 µL reactions containing 0.625 mM oligo dT primers and 1 µg RNA. Primers specific to human and mouse ZnT10, GAPDH and 18S rRNA (reference genes) were designed over intron/exon boundaries and are as listed in Table 2. Topoisomerase I (TOPI) was used as a further reference gene. TOPI primers were purchased from PrimerDesignLtd (UK). ZnT10 was subcloned into the vector pCR2.1-TOPO TA (Invitrogen). Standards for GAPDH and 18S rRNA products were generated by amplifying the region between the primers shown in Table 2, and subcloned into the vector pCR2.1-TOPO TA (Invitrogen). A TOPI PCR product was subcloned into the pGEM-T-easy vector to give the standard TOPI-pGEM. The identity of the products was confirmed by sequencing (MWG Biotech). Ten-fold serial dilutions of each of pCR2.1-hZnT10, pCR2.1-mZnT10, pCR2.1-GAPDH, pCR2.1-18S and TOPI-pGEM were used to generate standard curves. Primer pairs were used at a final concentration of 250 nM and amplification was carried out using the DNA Engine Opticon 2 (MJ Research) using Power SYBR Green PCR Master mix (Applied Biosystems) and thermal cycling parameters as in Table 2. Expression of the RNA of interest was calculated by taking the C_t_ value, averaging the duplicates, and then reading from the standard curve the arbitrary concentration of the samples. Efficiency values were calculated using the equation E = (10(−1/slope) - 1)*100 and were required to fall within the range of 80–115%. Values for ZnT10 were expressed relative to the value for the reference gene (GAPDH and TOPI for human samples; 18S rRNA and TOPI for mouse samples) measured in the same cDNA sample.

**Table 2:** Primers and cycling parameters for PCR^a^.

Name	Primer	Product size (bp)	Cycling parameters^a^
bZnT10	^844^ CGT AGC AGG TGA TTC CTT CAA C ^865^ ^956^ CAT CTC CCA TCA CAT GCA AAA G ^935^	140	95°C, 15 s annealing/extension 60°C, 10 s, 40 cycles
cMouse Znt10	^826^ GTA GCA GGT GAT TCC CTG AAC ^846^ ^965^ GTG ATG ACC ACA ACC ACG GAC ^945^	140	95°C, 15 s annealing/extension 60°C, 10 s, 40 cycles
dGAPDH^12^	^113^ TGA AGG TCG GAG TCA ACG GCT TTG ^136^ ^240^ CAT GTA AAC CAT GTA GTT GAG GTC ^217^	128	95°C, 15 s annealing/extension 60°C, 10 s, 40 cycles
e18S rRNA	^117^ CAT TAA GGG CGT GGG GCG G ^135^ ^247^ GTC GTG GGT TCT GCA TGA TG ^228^	131	95°C, 15 s annealing/extension 56°C, 10 s, 40 cycles

a An initial activation step of 95°C for 15 min. Times are given in the order denaturing, annealing extension, and the number of cycles is indicated.

b Numbered according to Genbank sequence NM_018713.

c Numbered according to Genbank sequence NM_001033286.

d Numbered according to Genbank sequence NM_002046.3.

e Numbered according to Genbank sequence NM_011296.

### Statistical Analysis

Experiments were performed in triplicate and each experiment was repeated at least twice. Results are expressed as a ratio of ZnT10 RNA: reference RNA, normalised to control samples. Data were tested for normality using the Shapiro-Wilk test. For normally distributed data differences between means were considered significant at p<0.05 using a two tailed Student's *t* test, taking into account homogeneity of variance. Braak scores and age were compared using non-parametric testing and the Mann Whitney U test. Correlations were carried out using Pearson's correlations and, where appropriate, Spearman's Rho correlation. Statistical analysis was carried out using Microsoft SPSS.

## Results

To investigate if there is any association between levels of ZnT10 expression in relevant regions of the brain and AD, we measured ZnT10 mRNA in frontal cortex of AD patients and controls and in frontal cortex of 12 month old female wild-type and APP/PS1 mice.

Demographic data for the human subjects studied are shown in Table 1. Full details for each subject can be found in Table S1.

Mean RNA Integrity numbers (RIN) were 5.32 (±1.88 S.D) with a range (from 2.5–7.3). These values indicate that, as expected RNA degradation had occurred in some of the post mortem brain tissue samples. For this reason all amplification products for analysis were less than 250 bp. Amplicons of this length have been shown to be independent of RNA quality [21]. Furthermore, we found no correlation (using Spearman's Rho correlation) between RIN and disease progression, (indicated by Braak score, p = 0.504). In addition, we found no significant correlations between RIN and PMD, pH and disease state (AD vs control) (p = 0.701, p = 0.676 and p = 0.845 respectively). Finally there was no correlation between RIN values and ZnT10 mRNA expression (p = 0.511).

**Figure 1:**
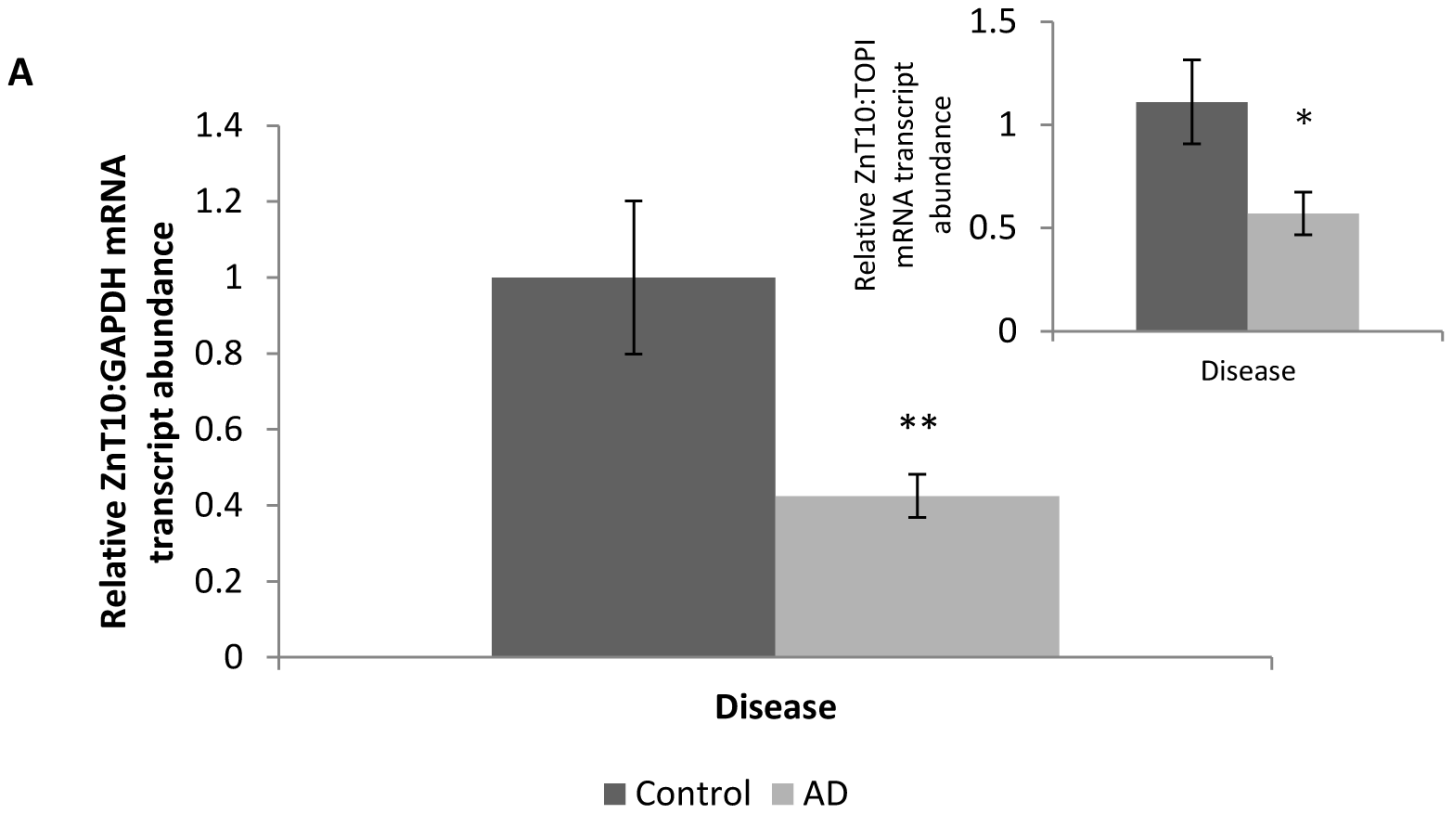
ZnT10 mRNA expression in human brain tissue. Relative levels of ZnT10 in brain tissue from AD brains (*n* = 16; 10 female) and control brains (*n* = 11; 4 female). Data are expressed relative to GAPDH mRNA levels measured in the same samples. Negative control RT-PCR reactions identical to those yielding the products shown except for the omission of Moloney murine leukemia virus reverse transcriptase resulted in no products. *Inset* data expressed relative to TOPI mRNA levels measured in the same sample. Primers are given in Table 1. All values are shown as mean ± SEM ** *p*<0.01, * *p*<0.05 by Student's *t* test.

**Figure 2:**
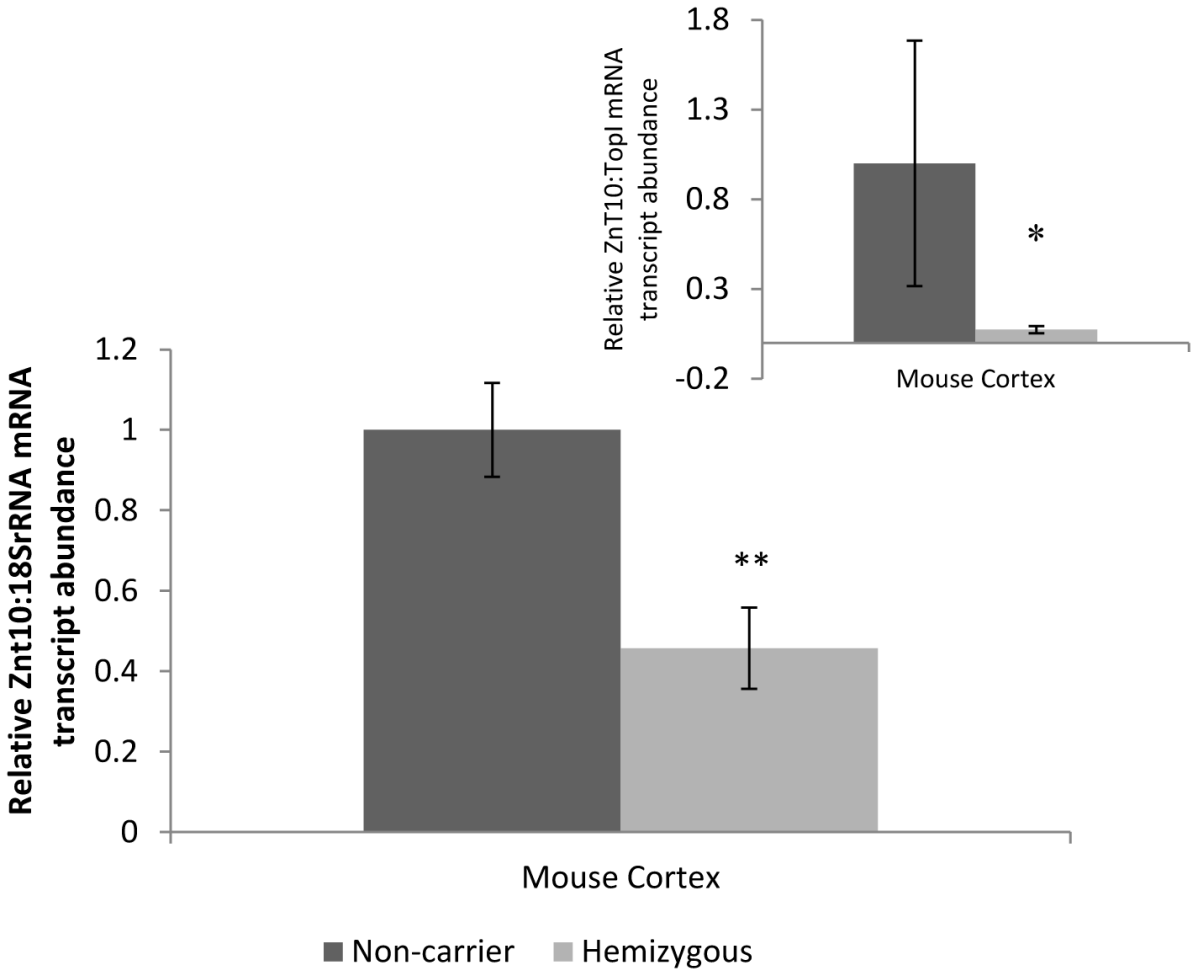
Znt10 mRNA expression in mouse brain tissue. Relative levels of Znt10 in brain tissue from APP/PS1 Tg mouse brains (*n* = 11) and control mouse brains (*n* = 5). Data are expressed relative to S18 rRNA mRNA levels measured in the same samples. Negative control RT-PCR reactions identical to those yielding the products shown except for the omission of Moloney murine leukemia virus reverse transcriptase resulted in no products. *Inset* data expressed relative to TOPI mRNA levels measured in the same sample. Primers are given in Table 1. All values are shown as mean ± SEM ** *p*<0.01, * *p*<0.05 by Student's *t* test.

There was no significant difference in age or post mortem delay (PMD) between control and AD subjects. Braak staging scores were significantly elevated (*P<*0.01) in AD patients (median = V) compared with control subjects (median = II). Compared with age-matched controls, we observed a significant decrease in ZnT10 mRNA in frontal cortex of AD patients (Figure 1). GAPDH and TOPI showed a consistent level of expression independent of patient diagnosis and were therefore chosen as the reference gene and normalisation control. There was no correlation between ZnT10 expression and Braak staging (*p* = 0.173) in the AD tissue. An indication that ZnT10 levels increase with age was observed, with a trend evident for controls (*p* = 0.088) and a near significant correlation in AD cases (*p = *0.066). When separated on the basis of sex, brain tissue from female AD patients revealed ZnT10 mRNA levels that correlated significantly with age (*p = *0.020). Correlation results are shown in Table 3. In the mouse samples, we observed a significant decrease in Znt10 mRNA levels in the APP/PS1-Tg mice compared with wild-type controls) (Figure 2). 18S rRNA and TOPI showed a consistent level of expression independent of mouse strain and were therefore chosen as the reference gene and normalisation controls.

**Table 3:** Correlation data examining the relationship between relative ZnT10 mRNA expression and human brain sample parameters.

Disease Group	Gender	Braak Stage	PMD	pH	Age
**Control**	Overall	0.772 (0.123)	0.170 (−0.471)	0.795 (−0.122)	0.088 (0.566)
	Male	0.741 (−0.205)	0.504 (−0.306)	0.873 (0.100)	0.148 (0.607)
	Female	0.333 (−0.866)	0.667 (−0.500)	-	0.667 (0.500)
**AD**	Overall	0.173 (−0.467)	0.123 (0.450)	0.204 (−0.395)	0.066 (0.525)
	Male	0.215 (−0.671)	0.391 (0.500)	0.624 (0.300)	0.935 (0.51)
	Female	0.425 (−0.331)	0.301 (0.419)	**0.023** * **(−0.778)**	**0.020** * **(0.790)**

For each correlation P values are stated with correlation coefficients in brackets. For Braak stages and when stratified for male and female data Spearman's Rho correlation have been carried out. For all other data Pearson's correlations are reported. – indicates insufficient data available.

* *p*<0.05.

## Discussion

We observed a significant decrease in ZnT10 mRNA in samples of frontal cortex from AD patients compared with age-matched controls, giving the first indication that ZnT10 regulation is affected in AD. Furthermore, there was some suggestion of a sex-specific effect; the lower levels of ZnT10 observed in female AD cases compared with controls was statistically significant (*P*<0.01 by Mann-Whitney U). Despite a 45% decrease in ZnT10 expression observed in the male AD cases compared with the controls, this did not reach statistical significance. As sample numbers were relatively small, larger numbers are required to draw robust conclusions from this finding. Also, there was no significant difference in ZnT10 mRNA levels between male and female control subjects (*p* = 0.66) or between male and female AD cases (*p = *0.67). The observed down regulation of a transporter of the ZnT family in AD is not without precedence. Znt3 mRNA was found to be reduced by up to 60% in all four cortical regions tested (medial temporal gyrus, superior occipital gyrus, superior parietal gyrus, and superior frontal gyrus) in AD cases compared with controls. Interestingly there was no significant differences in ZnT3 expression in the cerebellum [9].

In this study, only one area of the brain, frontal cortex, was available to examine ZnT10 expression and whilst this region had lower ZnT10 mRNA expression in AD patients, there are no data as yet to indicate if this effect is region-specific. Messenger RNA expression in brain tissue for other Zn transporters has also been shown to be dysregulated in AD. ZIP1, ZIP6, ZnT1 and ZnT6 - mRNAs were increased in the four cortical regions (middle temporal gyrus, superior occipital gyrus, superior parietal gyrus, and superior frontal gyrus), but not the cerebellum [22]. Further research is required to establish whether ZnT10 also follows this pattern.

There is evidence for a significant positive correlation between ZnT1 and Braak staging, which indicates NFT pathology. A trend towards a positive correlation has also been shown for ZnT6 and Braak staging [3], [4]. However, in the small number of cases analysed here there was no correlation between ZnT10 expression and Braak staging (*p* = 173) in the AD tissue. Intriguingly, however, there is an indication that ZnT10 levels increase with age, with a trend evident for controls (*p* = 0.088) and a near significant correlation in AD cases (*p = *0.066). When separated on the basis of sex, brain tissue from female AD patients revealed ZnT10 mRNA levels that correlated significantly with age (*p = *0.020). Details of the implications of age-related effects on ZnT expression have not been established, however it is known that there is a decline in Zn status with age (reviewed by [23], [24]). Whether the decline is related to inadequate diet (such as consumption of less red meat, rich in Zn) or a reduced ability of the body to maintain homeostasis of this micronutrient is still unclear.

In concordance with our findings in human subjects, we observed lower levels of Znt10 mRNA in frontal cortex from female APP/PS1 transgenic mice. APP/PS1 mice are a mouse model of AD containing two transgenes inserted at a single locus. The first is the APP sequence that is modified to encode the human Swedish mutations K595N/M596L. Secondly, PS1 corresponds to the human presenilin 1 gene. In this case there is a deletion of exon 9, therefore known as the DeltaE9 mutation (http://jaxmice.jax.org/strain/005864.html). As in AD, these mice have Aβ deposits and exhibit deficits in spatial learning [25]. The use of this mouse model to study AD is widespread, and in particular this model has been used to identify dysregulation of several members of the Znt family. Expression of Znt1, Znt3, Znt4, Znt5, Znt6 and Znt7 protein in the hippocampus and neocortex were higher in APP/PS1 mice than in controls, with only levels for Znt5 failing to reach significance [11]. Translation of results across species however must be applied with caution as data using this mouse model revealed an increase in Znt3 protein levels, whereas in human AD brain a decrease in both ZnT3 mRNA and protein levels was observed [9], [10].

Other studies on the expression of Zn transporters in transgenic mouse models of AD have investigated the distribution within senile plaques by immunohistochemistry. Intriguingly distinct expression patterns were uncovered, with Znt1 and Znt4 being detected throughout the senile plaques whereas Znt3, Znt5 and Znt6 were only associated with peripheral regions, and Znt7 was detected predominantly in the core [11]. Our current data do not indicate the location of Znt10 within mouse brain tissue, nor where the differences between controls and APP/PS1 mice lie, and further study towards obtaining higher-resolution data of this type is warranted.

Human studies of ZnT dysregulation in AD have highlighted the importance of specifying the stage of disease progression. There are differences in ZnT expression profiles between AD and the precursor states described in the literature. ZnT1 was found to be decreased significantly in the hippocampus of subjects with the precursor states MCI (a stage from which 95% of progress to develop dementia) and pre-clinical AD (PCAD; where subjects have no overt clinical manifestations of AD but pathology is revealed post-mortem), as well as in AD [3], [4]. Both ZnT4 and ZnT6 were elevated in PCAD [4] and in the hippocampus of AD patients [5], however there was no significant difference in levels of either transporter between MCI and age-matched controls. The role of ZnT10 in the progression of AD will be an important area to address in future research, which should elucidate its level of expression in both PCAD and MCI. Our results thus far suggest a mechanism for ZnT10 dysregulation in the final disease state of AD. We have previously shown that under basal conditions ZnT10 is localised to the Golgi apparatus in a neuroblastoma cell line model, and is translocated towards the plasma membrane on the addition of extracellular (100 µM) Zn concurrent with a down-regulation of ZnT10 at both the mRNA and protein levels [17]. Therefore it is possible that the increase in Zn reported in AD brain [26] induces a down-regulation of ZnT10 mRNA, as observed in this study. Moreover, the localisation of ZnT10 suggests two possible mechanisms through which increased expression could exacerbate AD progression. Initially, localisation to the Golgi apparatus may promote Aβ formation by increasing Zn sequestration in this organelle thereby enabling the binding of Zn to APP and the inhibition of α-secretase, in a similar manner to that hypothesised for ZnT6 [4]. Secondly, as elevated levels of Zn induce the translocation of ZnT10 towards the plasma membrane it may act in a similar manner as has been suggested for ZnT1 [4], effluxing Zn into the extracellular space to providing Zn ions for initiation of Aβ deposition and senile plaque formation.

Recent observations identifying frame-shift mutations in the *SLC30A10* gene in patients with the neurological conditions dystonia and Parkinsonism that lead to hypermanganesemia in brain regions [18] alongside rescue experiments performed in a yeast mutant demonstrating the ability of wild-type ZnT10 to restore growth in a Mn sensitive yeast mutant *Δpmr1* [20] indicate that ZnT10 may act as a Mn transporter. As these experiments have not been carried out in the presence of other metals, or by a more direct methodology (e.g. *Xenopus laevis* oocyte system) the precise specificity of this transporter remains unknown, but the results of the current study should nonetheless be interpreted with cognisance of the likelihood that ZnT10 transports manganese. Given the known link between neurological conditions and chronic exposure to Mn, it is possibile that the dys-homeostasis of ZnT10 observed in the AD brain is related to changes in levels of Mn rather than Zn., Mechanisms of Mn-induced neurological changes remain to be elucidated, although oxidative stress, protein aggregation or altered homeostatic conditions of other divalent metals that share similar transport mechanisms have been suggested [13].

Overall we uncover, for the first time, changes in ZnT10 mRNA levels in a vulnerable region of AD brain, which is paralleled in the AD transgenic mouse brain. Given the subcellular localisation and predicted function of ZnT10 as both a Mn and Zn transporter it is possible that tdysregulation of these metals could contribute to increased Aβ deposition and senile plaque formation and ultimately disease progression.

## Supporting Information

Table S1 Individual data for each subject including individual actual and normalised ratios for each RT-qPCR. – indicates PCR failed quality checks and therefore sample not used in final analysis.
(DOCX)
