# Supplementary material for: Altered Expression of ZnT10 in Alzheimer's Disease Brain
Source: PLoS One. 2013 May 31;8(5):e65475. doi: 10.1371/journal.pone.0065475 (PMC3669266; doi:10.1371/journal.pone.0065475)
Supplement: Table S1 — Individual data for each subject including individual actual and normalised ratios for each RT-qPCR. – indicates PCR failed quality checks and therefore sample not used in final analysis. (DOCX) [file pone.0065475.s001.docx]

| **Diagnosis** | **Age** | **Gender** | **PM delay** | **Braak stage** | **CSF pH** | **ZnT10:GAPDH ratio** | **Normalised ZnT10:GAPDH ratio** | **ZnT10:TOP1 ratio** | **Normalised ZnT10:TOPI ratio** |
| --- | --- | --- | --- | --- | --- | --- | --- | --- | --- |
| AD | 76 | f | 6 | 5 | 6.5 | 4.35E-04 | 0.30 | 1.35E-09 | 2.32 |
| AD | 84 | f | 29 | NA | 6.1 | 1.06E-03 | 0.74 | 1.24E-10 | 1.04 |
| AD | 71 | m | 24 | 5 | 5.9 | 5.49E-04 | 0.38 | 1.48E-10 | 1.41 |
| AD | 78 | m | 20 | 4 | 5.97 | 8.67E-04 | 0.60 | 2.30E-10 | - |
| AD | 80 | f | 4 | NA | 6.1 | 8.14E-04 | 0.57 | 1.10E-09 | 1.68 |
| AD | 88 | f | 16 | 3 | 5.92 | 1.24E-03 | 0.86 | 6.27E-10 | - |
| AD | 79 | f | 15 | 5 | 6.58 | 3.73E-04 | 0.26 | 7.89E-10 | 0.71 |
| AD | 79 | f | 12 | 4 | 7.3 | 3.15E-04 | 0.22 | 7.03E-10 | 0.14 |
| AD | 68 | m | 16 | 5 | 6 | 7.70E-04 | 0.54 | 3.34E-11 | - |
| AD | 74 | m | 15 | NA | 5.78 | 2.48E-03 | - | 1.22E-09 | 0.74 |
| AD | 91 | f | 17 | 4 | 5.76 | 2.30E-03 | - | 4.44E-11 | 0.84 |
| AD | 77 | f | 8 | NA | 6.16 | 3.19E-04 | 0.22 | 8.66E-10 | - |
| AD | 74 | m | 6 | 5 | 5.78 | 2.25E-04 | 0.16 | 6.21E-10 | 0.12 |
| AD | 71 | m | 19 | 6 | 6.14 | 3.89E-04 | 0.27 | 1.73E-09 | 0.15 |
| AD | 83 | f | 16 | 2 | NA | 5.69E-04 | 0.40 | 1.03E-09 | 0.23 |
| AD | 72 | f | 24 | NA | 6.04 | 5.00E-03 | - | 3.67E-09 | 1.08 |
| Con | 80 | m | 30 | 4 | 6.23 | 4.67E-04 | 0.33 | 2.35E-09 | 0.62 |
| Con | 72 | m | 31 | 1 | 6.25 | 3.40E-04 | 0.24 | 1.05E-09 | 0.78 |
| Con | 57 | m | 21 | NA | 6.57 | 1.13E-03 | 0.79 | 1.43E-09 | 0.69 |
| Con | 84 | f | 7 | 4 | 6.55 | 1.63E-03 | 1.14 | 5.32E-11 | 0.03 |
| Con | 65 | m | 17 | 0 | NA | 1.89E-03 | 1.32 | 1.71E-09 | 1.20 |
| Con | 80 | f | 12 | 2 | NA | 2.15E-03 | - | 2.90E-11 | 0.04 |
| Con | 81 | f | 32 | NA | 6.23 | - | 1.50 | 7.21E-10 | 0.85 |
| Con | 93 | m | 10 | 3 | 6.27 | 3.24E-03 | 2.26 | 1.46E-10 | 0.61 |
| Con | 82 | m | 36 | NA | NA | 1.92E-03 | 1.34 | 1.15E-10 | - |
| Con | 19 | m | 17 | 0 | 6.9 | 4.25E-04 | 0.30 | 7.54E-10 | 1.01 |
| Con | 75 | f | 16 | 4 | 5.57 | 1.15E-03 | 0.80 | 8.49E-10 | - |

**Supplementary Table S1: Individual data for each subject.** – indicates PCR failed quality checks and therefore sample not used in analysis
